# Cellular senescence, rejuvenation and potential immortality

**DOI:** 10.1098/rspb.2021.2434

**Published:** 2022-03-09

**Authors:** A. Rupert Sheldrake

**Affiliations:** Schumacher College, Totnes, UK

**Keywords:** cellular senescence, cellular rejuvenation, damaged cell constituents, asymmetric cell division, cancer cells, stem cells

## Abstract

Ageing, death, and potential immortality lie at the heart of biology, but two seemingly incompatible paradigms coexist in different research communities and have done since the nineteenth century. The universal senescence paradigm sees senescence as inevitable in all cells. Damage accumulates. The potential immortality paradigm sees some cells as potentially immortal, especially unicellular organisms, germ cells and cancerous cells. Recent research with animal cells, yeasts and bacteria show that damaged cell constituents do in fact build up, but can be diluted by growth and cell division, especially by asymmetric cell division. By contrast, mammalian embryonic stem cells and many cancerous and ‘immortalized’ cell lines divide symmetrically, and yet replicate indefinitely. How do they acquire their potential immortality? I suggest they are rejuvenated by excreting damaged cell constituents in extracellular vesicles. If so, our understanding of cellular senescence, rejuvenation and potential immortality could be brought together in a new synthesis, which I call the cellular rejuvenation hypothesis: damaged cell constituents build up in all cells, but cells can be rejuvenated either by growth and cell division or, in ‘immortal’ cell lines, by excreting damaged cell constituents. In electronic supplementary material, appendix, I outline nine ways in which this hypothesis could be tested.

## 1. Introduction

Since the late nineteenth century, there have been two principal schools of thought about cellular ageing. One sees senescence as universal. Wear and tear are inevitable. The problem is to explain rejuvenation, without which all cells would die out. The other paradigm assumes that some cells are potentially immortal and immune to senescence, namely unicellular organisms, the germ cells of multicellular organisms and ‘immortal’ cell lines grown in laboratories. The problem is to explain senescence in the somatic cells of multicellular organisms. If senescence is optional, why did it evolve?

This debate was epitomized in the conflicting views of Émile Maupas (1842–1916) and August Weismann (1834–1914) and is still unresolved. Maupas, a French zoologist, argued that senescence is inherent in all cells, and that rejuvenation depends on sexual processes [1]. Weismann, a German professor of biology, proposed that unicellular organisms and germ cells are potentially immortal, but that somatic cells of multicellular organisms senesce. He assumed that animal germ cells inherited their potential immortality from unicellular organisms, which, in his own words, ‘carry the potentiality of unending life’. He continued, ‘The reproductive cells cannot lose that capacity for unlimited reproduction … But the somatic cells have lost this power… they became restricted to a fixed, though perhaps very large number of cell generations' [2].

These two approaches, which I call the ‘universal senescence paradigm’ (USP) and the ‘potential immortality paradigm’ (PIP), are still widely influential, but in different areas of biology. Within the last 15 years, the study of senescence and rejuvenation in bacteria and yeasts has led some researchers to argue that senescence through the accumulation of damage is inevitable in all forms of life, including unicellular organisms [3,4]. The origins of ageing and the origins of life itself may be intertwined [5].

By contrast, PIP is still taken for granted by many researchers on germ cells, embryonic stem cells (ESC) and cancer. This assumption is explicit in Kirkwood's [6] influential ‘disposable soma hypothesis', explicitly based on Weismann's ideas, which assumes that germ cells retain their youthfulness by virtue of energetically costly repair systems. Multicellular animals have evolved to allocate fewer resources to the repair and maintenance of somatic cells, which eventually die anyway. Error regulation is a ‘luxury’ that somatic cells cannot afford [7,8].

Both USP and PIP agree that damaged cell constituents (DCC) accumulate in somatic cells of multicellular animals. They disagree in that USP assumes that damage accumulates in all cells, whereas PIP assumes that some cells have almost infallible repair systems that are downregulated in normal somatic cells to save resources.

Here, I argue that recent advances in cell biology may enable this longstanding debate to be resolved through an expanded concept of rejuvenation.

I first look at cellular damage and repair systems at the molecular level, and then explore senescence, rejuvenation and potential immortality in all kingdoms of life, starting with multicellular animals, followed by plants, then bacteria and yeasts which, although they are unicellular organisms, are not immune to senescence, as Weismann assumed. I then discuss animal egg cells. Do they have near-perfect repair systems, as PIP supposes?

Finally, I consider the puzzle posed for USP by ‘immortal’ mammalian cell lines, including ESC and cancerous cells, such as the famous HeLa cells, initially isolated from the cervical cancer of an African-American woman, Henrietta Lacks, in 1951 [9] and still multiplying by symmetrical cell division in laboratories around the world. How have they acquired this immortality? Many cancers and ESC are now known to excrete large numbers of extracellular vesicles. I suggest that these cells rejuvenate themselves by excreting DCC in extracellular vesicles, an ability that most other animal cells lack. This hypothesis may provide a way of reconciling USP and PIP and point toward a unified understanding of senescence and rejuvenation applicable to all forms of life, which I call the cellular rejuvenation hypothesis. In electronic supplementary material, appendix, I outline nine ways in which this hypothess could be tested experimentally.

## 2. The repair of cellular damage

Some of the principal causes of molecular damage in cells are reactive oxygen species (ROS), highly reactive chemicals, such as peroxides, produced as a result of oxidative phosphorylation in mitochondria and other oxidative processes. ROS damage lipids by peroxidation, and proteins by carbonylation, introducing ketone or aldehyde groups into protein sidechains [10,11]. Lower levels of ROS generally lead to less accumulation of age-related damage [12]. This seems to be the main reason why caloric restriction in the diets of a wide variety of organisms, including yeasts, nematode worms, fruit flies, rodents and primates, prolongs lifespan and reduces the incidence of age-related diseases [13].

In addition to oxidative damage, various kinds of stress, including heat stress, lead to the unfolding of proteins, followed by misfolding and aggregation, for example in ‘stress granules’ made up of denatured ribonucleoproteins that accumulate when protein synthesis stalls [14].

Oxidative and stress damage are often reversible. Most carbonylated, misfolded and dysfunctional proteins are degraded by the ubiquitin–proteasome system, which is highly conserved in eukaryotic organisms. The first step is the tagging of misfolded or damaged proteins by covalent bonds to ubiquitin, a small protein found in almost all eukaryotic cells. These tags identify the damaged proteins as candidates for digestion by proteasomes, complex structures with tunnel-like internal cavities capable of degrading hundreds of different proteins [15].

Some aggregates of denatured proteins can be dissolved; their proteins are ubiquinated and degraded by proteasomes [16]. However, some resist disassembly and like other persistent aggregates can be dissolved only after being incorporated into autophagosomal vesicles [17], which have double membranes, and form around denatured protein complexes, dysfunctional mitochondria and other damaged structures, segregating the material from the rest of the cytoplasm. These vesicles fuse with lysosomes, whose hydrolytic enzymes digest most of the vesicular contents for recycling [18].

Damage to membranes can be overcome by several different repair systems [19]. Although protein, membrane and other repair systems [20] are highly effective, they are not infallible, at least in the somatic cells of animals. DCC accumulate as time goes on, sooner or later resulting in cellular senescence [21–23]. DCC include indigestible protein aggregates, defective mitochondria and lipofuscin granules, also known as ‘age pigment granules', which accumulate in lysosomes as ‘residual bodies’ [24,25]. These are all hallmarks of ageing, and increase with age in the cells of many mammalian tissues with harmful effects [26,27]. The accumulation of indigestible residues is exaggerated in pathological conditions like Alzheimer's disease, where denatured protein aggregates accumulate within neurons and plaques of denatured amyloid peptide build up between them [28].

Although some effects of ageing are reversible, the accumulation of DCC is irreversible. If cells stop growing and dividing, DCC continue to accumulate not only in mammalian cells, but in nematode worms, insects [29], plants [30], and in many other kinds of organisms.

In summary, not all cellular damage can be repaired. Cells can avoid senescence only if they get rid of DCC. How can they do so? I look first at the strategies employed by somatic cells in multicellular animals.

## 3. Cellular rejuvenation in multicellular animals

If somatic cells accumulate DCC as they grow older, how are some cells, especially stem cells, rejuvenated?

**Figure 1.**
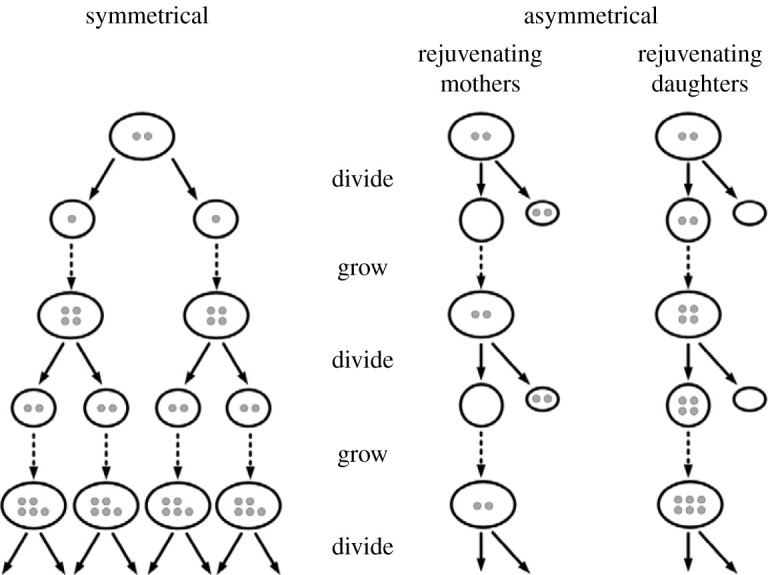
The distribution of damaged cell constituents (DCC) in symmetrical and asymmetrical cell division. DCC (small grey circles) build up in cells as time goes on and are inherited by the next generation through cell division. In symmetrical division they are distributed more or less equally between sisters. In asymmetrical division, the mother can be rejuvenated at the expense of the daughter, as in epithelial stem cells; or the daughter can be rejuvenated at the expense of the mother, as in budding yeasts.

In an article in *Nature* entitled ‘The ageing, growth and death of cells' [31], I proposed the hypothesis that some cells escape senescence because they dilute DCC by growing and dividing. If they divide symmetrically, both daughters inherit similar amounts of DCC. If they divide asymmetrically and one daughter inherits most DCC, the other is rejuvenated, in the sense that it is freed from DCC (figure 1). Cell populations can grow quicker by symmetrical cell division, but DCC build up within them unless they keep on dividing fast enough. Mathematical models based on this hypothesis show a variety of outcomes depending on the rates of accumulation of DCC, the rates of cell division, the degree of asymmetrical partitioning of DCC, and the degree to which damage accumulation is autocatalytic. In symmetrically dividing cells, if DCC accumulate faster than cell division dilutes them, cell lines die out [32,33].

Since around 2005 much evidence has built up for the asymmetrical partitioning of DCC in asymmetrical cell division (ACD) in animal stem cells [34], and also in bacteria and yeasts, as discussed below.

### (a) Stem cells

Stem cells usually divide asymmetrically and one of the ensuing cells is rejuvenated at the expense of the other. In adults, stem cells themselves are continually rejuvenated as they produce a succession of mortal daughters. In embryos, some stem cells work the other way round: their progeny are rejuvenated while the stem cells age, a pattern also seen in bacteria and yeasts, as discussed below (figure 1).

The stem cells in our own skin, bone marrow and intestinal crypts are continually rejuvenated, while their progeny differentiate and die [35,36]. In a study on people suffering from a degenerative disease in which mutant proteins accumulate in insoluble aggregates, the intestinal stem cells were free of these inclusions, even in elderly patients, while their short-lived progeny contained large amounts [37].

Stem cells are not entirely immune from ageing; some senesce as a result of genetic mutations, epigenetic changes and environmental damage [38]. When stem cells are killed by damage, nearby stem cells divide symmetrically to replace them [39], losing the rejuvenative advantage of ACD.

### (b) The partitioning of damaged cell constituents during asymmetric cell division

DCC aggregate in several ways. Denatured proteins accumulate in bodies near the nucleus, variously called aggresomes or JUxta-Nuclear Quality (JUNQ) control compartments. Meanwhile, Insoluble Protein Deposits (IPODs) [40] form in the peripheral cytoplasm.

One way in which DCC are differentially partitioned during ACD is through the linkage of aggresomes to one of the centrosomes of a dividing cell, resulting in their asymmetric distribution during cell division [41,42]. IPODs are partitioned asymmetrically by a different cytoskeleton-based system from aggresomes and JUNQs [34,43]. Damaged mitochondria are also partitioned asymmetrically into the daughter cells [44,45].

The result of these processes is that stem cells are rejuvenated and their daughters inherit ‘cargoes’ of DCC.

### (c) The 'Hayflick limit’ in symmetrically dividing cells

Weismann's idea about somatic cells having a limited capacity to divide was supported by the study of mammalian cells in tissue culture, such as fibroblasts, which divide symmetrically and die out after a limited number of divisions, sometimes called the ‘Hayflick limit’, which rarely exceeds 50–60 divisions [46,47]. At first sight, the senescence of these cell lines, which divide slower as they approach the limit of their lifespan, seems to support the idea of a build-up of DCC. So does the fact that oxidative stress accelerates their senescence [48]. However, DCC alone cannot explain the senescence of these lines, which also depends on the shortening of telomeres [49].

Telomeres are structures formed of repetitive DNA sequences at the ends of chromosomes, linked to a protein complex called shelterin [50]. In embryos, cells start with long telomeres which are usually shortened through successive divisions. Through a pre-programmed ‘count-down’ process, cell division stops when telomeres are too short. This system may have evolved in part to help prevent somatic cells proliferating cancerously [51]. Telomere-dependent ageing can be reversed if the telomerase enzyme system is activated and lengthens the telomeres, as occurs in ESC, some adult stem cells and in many cancer cells, enabling cell division to continue [52,53]. However, the effects of genetic manipulation may involve not only a lengthening of telomeres but also the activation of the c-*Myc* oncogene [54], which is a ‘master regulator’ of genetic changes characteristic of cancerous cells [55].

The reversibility of telomere-centred ageing seems to support PIP. So does another kind of epigenetic ageing, in which DNA and associated histone proteins are methylated progressively, making the cells less prone to divide as time goes on. The contrary process, demethylation, has a rejuvenating effect [56]. DNA and histones are demethylated in many cancers [53,57].

I return to a discussion of immortalized cell lines and cancer cells in §5.

## 4. How are cells rejuvenated in plants?

Whereas multicellular animals stop growing, senesce and die, some plants grow indefinitely, and their growing tips do not senesce. For example, trembling aspen trees propagate themselves vegetatively by root suckers forming clonal groves, some of which are more than 10 000 years old [58]. Some crops, like potatoes, are routinely propagated vegetatively.

All vascular plants, including ferns, conifers and flowering plants, contain meristems in their shoot and root tips, which are regions in which cells divide, and from which the stems, leaves, flowers, fruits and roots are derived. In the heart of these meristems are stem cells that divide by ACD. Their daughters undergo further divisions within young roots and shoots, then differentiate and sooner or later die [59].

ACD has been studied in the model plant *Arabidopsis thaliana* [60], but little is yet known about the partitioning of DCC. However, in the light of research on animal stem cells, bacteria and yeasts it seems likely that the stem cells are rejuvenated by ACD, while their mortal daughters inherit DCC. If so, plant stem cells would fit into a pattern of cellular senescence and rejuvenation found in other realms of life. This is a testable hypothesis, as discussed in electronic supplementary material, appendix.

### (a) Multiple fission

In many species of unicellular algae, including *Chlorella*, division takes place by multiple fission. A mother cell divides repeatedly to form 4, 8, 16 and up to 128 daughter cells [61,62] depending on the growth conditions [63], necessarily diluting DCC.

Many algae and plants, including mosses and ferns, reproduce vegetatively by spores, which are often produced in vast numbers by multiple fission. For example, in the sporangia on the fronds of the fern *Marattia*, each archesporial cell, itself produced by ACD, can give rise to 2656 spores [64]. Many fungi and protozoa also produce spores by multiple fission.

The patterns of cell division in plants are consistent with the idea that DCC build up in all cells over time, but some cells can be rejuvenated by ACD or by the dilution of DCC by multiple fission. Plants fit well within USP. Indeed, Weismann's PIP never applied to plants in the first place, because germ cells are not separated off from somatic cells at an early stage of embryology. Flowers develop from meristems in shoots.

## 5. Rejuvenation in bacteria and yeasts

Over the last 15 years there has been a widespread recognition that many bacteria senesce and undergo rejuvenation through growth and cell division [4,65]. Rejuvenation generally depends on ACD, even in cells that appear to divide symmetrically. The same is true of yeasts.

### (a) Bacteria

**Figure 2.**
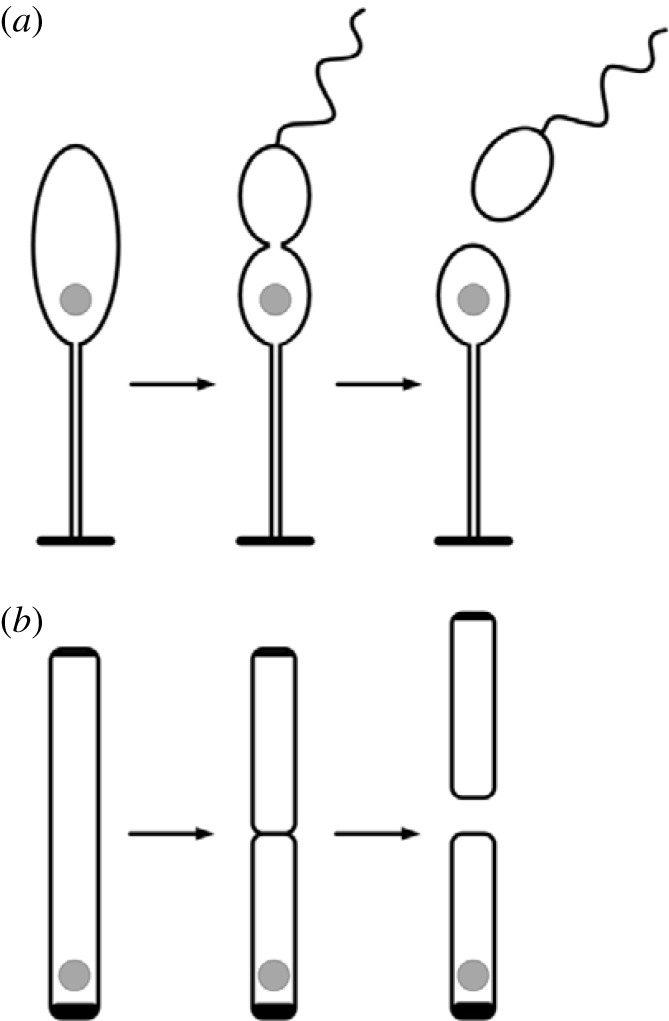
The partitioning of DCC in bacterial cell division. (*a*) A *Caulobacter* cell attached to a solid substratum by its stalk. When it divides DCC (grey circle) remain within the stalked cell, while its rejuvenated daughter, a swarmer cell, swims away, then settles down and becomes a stalked cell itself. Stalked cells divide repeatedly, accumulate DCC, and eventually die. (*b*) *E. coli* appears to divide symmetrically, but an aggregate of DCC located near the old pole of the cell is asymmetrically partitioned into the cell that inherits the old pole, while the other is rejuvenated.

**T**he aquatic bacterium *Caulobacter crescentus* has two kinds of cells: stalked cells rooted to a solid substratum through a tube-like stalk, and flagella-bearing swarmer cells (figure 2). Only stalked cells divide; at first, they give rise to swarmers in rapid succession, then produce them slower and slower, until they finally stop dividing and die. Through ACD, the stalked cells retain DCC and undergo a form of replicative ageing while the swarmer cells are rejuvenated [66].

Although most bacteria appear to divide symmetrically, this appearance is deceptive. The bacterium *Escherischia coli* divides into equal-sized daughters, but one inherits an old cell wall while the other receives a new one (figure 2). Daughters that inherit the old poles grow slower, produce fewer offspring and are more likely to die [67]. Aggregates of denatured proteins usually accumulate near the old pole. Sometimes new-pole cells inherit these inclusion bodies instead, and then they too divide slower [68]. In most cells that divide rapidly, there are no visible denatured protein aggregates, but cells with the old pole still seem to inherit DCC and divide slower than their sisters [69,70]. Thus in *E. coli* as in *Caulobacter*, one daughter cell is rejuvenated at the expense of the other.

### (b) Yeasts

Budding yeasts divide asymmetrically by budding off small daughter cells, which then grow and become mother cells themselves. In a classic study of the budding yeast *Saccharomyces cerevisiae*, used in brewing and baking for millennia, Mortimer & Johnston [71] found that mother cells gave rise to an average of 24 daughter cells; the rate of division slowed toward the last generation, and then the mother cells died.

Recent research on budding yeasts has revealed more details about the retention of denatured protein aggregates [72] and ageing mitochondria [73,74] by the mother cells while the daughters are freed from them. The cytoskeleton plays an essential role [75], as does the endoplasmic reticulum [76].

Unlike budding yeasts, fission yeasts, like *Saccharomyce pombe*, appear to divide symmetrically. However, as in *E. coli*, DCC in the form of protein aggregates are retained by one daughter cell while the other is freed from them and subsequently divides faster [33]. Under favourable growth conditions, in which cells grew and divided rapidly, few protein aggregates accumulated; many of the cells showed no signs of ageing, but a minority inherited denatured protein aggregates and died [77].

Yeasts are now among the most important model organisms for studies of senescence and rejuvenation at the cellular level [4,78]. Weismann's assumption that unicellular organisms are immune to senescence is not true, at least in the case of the most-studied yeasts and bacteria.

## 6. Are animal germ cells immune to senescence?

In PIP, there is no need to explain the rejuvenation of animal germ cells. They are immune to senescence *ex hypothesi*. By contrast, USP suggests that DCC are likely to accumulate in germ cells as they do in other cells. If so, how are germ cells rejuvenated?

Male gametes do not need to be rejuvenated. They are usually short-lived, and their cytoplasm plays little or no role in the new organism. In both animals and plants, they are formed by symmetrical cell divisions. In humans and other mammals, primary spermatocytes divide by meiosis to produce four viable sperm cells [79]. In flowers, the pollen mother cells divide by meiosis and give rise to four viable pollen grains [80].

By contrast, eggs are formed by ACD in both animals and plants [31]. They are surrounded by dying sisters. For example, in the fruit fly *Drosophila*, a cell called the oogonium goes through four cycles of cell division to produce 16 cells, of which only one, the oocyte, gives rise to the egg; the others, called nurse cells, nourish and sustain it and die [81]. The oocyte then undergoes meiosis, producing the egg and short-lived polar bodies [82].

In plants, the sisters of maturing egg cells die, just as they do in animals. For example, in the flowers of *Arabidopsis*, the megasporocyte, the cell from which the egg is ultimately derived, produces four cells by meiosis, three of which die [83].

To my knowledge, the partitioning of DCC in the production of egg cells in animals and plants has not yet been studied, nor has their partitioning between the oospheres and periplasm of fungi (see in electronic supplementary material, appendix).

## 7. How do ‘immortal’ cell lines and cancer cells avoid senescence?

PIP seems to be confirmed by ‘immortal’ mammalian and human cell lines that are not subject to the Hayflick limit, such as HeLa cells. Hundreds of immortal cell lines are commercially available and routinely used in research. Most show one or more of the hallmarks of cancer, including the activation of telomerase, enhanced energy production by glycolysis [84], and genetic changes, including increased mutation rates and chromosomal instability [85].

In cancers, not only do the cells escape from the controls that stop normal cells from dividing excessively, but they also need to avoid senescence, or else they would die out. One way in which they are protected from the build up of DCC is by a shift in energy production toward glycolysis, a feature of cancerous cells noted more than 80 years ago by the biochemist Otto Warburg and known as the Warburg effect [85]. Glycolysis produces fewer ROS than oxidative phosphorylation and hence less oxidative damage.

In some cancers, cells are rejuvenated by ACD in a similar way to normal stem cells; indeed many cancers arise from stem cells in the first place, and also contain stem cells that divide by ACD [86,87]. However, some cancerous cells grown *in vitro* divide symmetrically, like HeLa cells, without the kind of cryptic asymmetry that occurs in the divisions of *E. coli* and fission yeast cells ([88], their electronic supplementary material, figure S1).

This is where the paradigms come into head-on collision. For PIP, immortal cell lines pose no problem; they have near-perfect repair systems *ex hypothesi*. By contrast, these cell lines are problematic for USP. How can cancer cell lines that divide symmetrically and are not rejuvenated by ACD escape from senescence? The only possible answer seems to be that they have another way of getting rid of DCC, namely by excreting them in vesicles [31].

**Figure 3.**
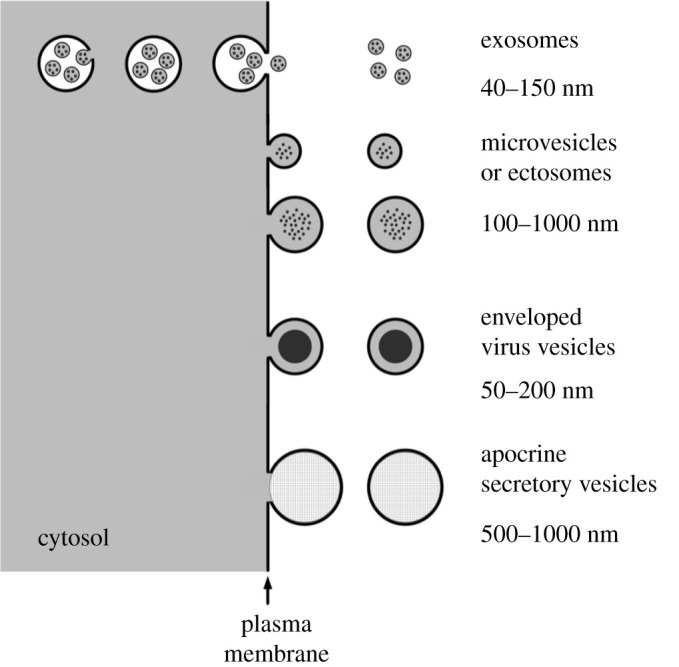
Some of the different kinds of membrane-bound extracellular vesicles secreted from mammalian cells. Exosomes are first budded off into intracellular vacuoles, called multivesicular bodies, before being released into the extracellular fluid when the multivesicular bodies fuse with the plasma membrane. Microvesicles of various types, also called ectosomes, are budded off the plasma membrane directly into the extracellular fluid [89,90]. Vesicles containing viruses, such as human immunodeficiency virus (HIV), are released within an envelope derived from the cell membrane [91]. Some glandular cells secrete vesicles, like the membrane-bound lipid globules produced by mammary glands as constituents of milk [92]. In addition, when dying cells break up they release membrane-bound ‘apoptotic bodies’ of varying sizes [90].

Many types of animal cell are now known to bud off membrane-bound vesicles into the extracellular space (figure 3). Some of these vesicles play an important role in intercellular communication, transferring DNA, RNA, proteins, lipids and virus particles from cell to cell [93]. Extracellular vesicles can be taken up by other cells either by fusing with their membranes, thus transferring their contents to the cytoplasm of the receptor cell, or by being engulfed by phagocytotic cells [94].

Some extracellular vesicles are known to contain DCC, including damaged DNA [95] and damaged proteins [89], including misfolded amyloid peptide aggregates produced in neurons affected by Alzheimer's disease [96,97]. Differentiating erythrocytes shed unwanted proteins in exosomes [98].

Many types of cancer cell release extracellular vesicles [93], and some amoeboid cancer cells shed unusually large ones, called oncosomes [99]. Extracellular vesicles carried in the blood are now used as diagnostic biomarkers for a wide range of cancers [100]. The infection of cells with cancer viruses changes the types of extracellular vesicles they produce [101], and the transformation from normal cells to immortalized cancer cells leads to a striking increase in the secretion of extracellular vesicles [102]. Vesicles from HeLa cells contain unfolded proteins, among other molecules [103].

A comparable process occurs in bacteria. Many species produce ‘minicells’ that lack chromosomes, cannot proliferate, and die. In *E. coli*, minicells are budded off from cell poles and contain aggregates of damaged proteins. Bacteria are rejuvenated by this ‘damage disposal mechanism’ and their daughters divide faster as a result [4,104]. This is a very underexplored area if research, and in electronic supplementary material, appendix, I suggest several new lines of enquiry.

## 8. How do embryonic stem cells escape senescence?

Mammalian ESC are capable of developing into any type of cell in the body. When cultured *in vitro* they divide symmetrically and are potentially immortal; not subject to the Hayflick limit [105]. They are the only normal cells that divide symmetrically and share potential immortality with immortalized and cancerous cell lines.

Under natural conditions, ESC are a short-lived phase at the beginning of embryology.

They are difficult to maintain *in vitro* under standard atmospheric conditions because they tend to differentiate spontaneously. They are best grown at oxygen levels of 2–5%, way below the usual 21% in the atmosphere [106,107]. These conditions resemble their hypoxic environment within pre-implantation embryos [108], which may protect them from the build-up of DCC by reducing damage from ROS. ESC also derive much of their energy from glycolysis, further reducing oxidative damage.

ESC prolifically produce extracellular vesicles, and vesicles derived from cultured ESC are now being tested for their therapeutic potential as a new version of stem cell therapy [109,110].

Four major marker genes are highly expressed in ESC, including c-*Myc*, each of which then activates gene complexes or modules. In many types of cancer, at least one of these ESC marker genes is activated, especially in the most aggressive tumours [111,112]. Although genes such as c-*Myc* are usually called oncogenes, as if they are specific to cancer, this is misleading. Cancer cells seem to reactivate key features of quintessentially youthful ESC. When cancer cells are grown under hypoxic conditions, like those under which ESC have evolved, further ESC genes are often activated and the cells become more prone to form aggressive tumours [113,114].

I suggest that one of the abilities that may be present in ESC and regained by cancerous cell lines may be the ability to excrete DCC in extracellular vesicles. In normal development, this ability is suppressed as cells differentiate. If somatic cells excreted DCC, intercellular spaces would become like open sewers, overwhelming the ability of phagocytes to clean up the debris. However, this excretory ability may be reactivated in immortalized cell lines and some cancers.

Thus many of the features of immortal cell lines and cancer cells may arise as a result of re-activating ESC gene modules. This similarity may include a rejuvenative system that enables the cells to get rid of DCC in vesicles.

## 9. Conclusion

Both USP and PIP are compatible with the dilution of DCC by growth and cell division, especially by ACD, in animals, yeasts and bacteria. Both paradigms predict similar processes in plants. Both paradigms are consistent with the dilution of DCC by multiple fission in plants, algae, fungi, protozoa and bacteria.

However, the paradigms conflict in relation to ESC and symmetrically dividing immortalized cell lines. USP assumes that DCC inevitably accumulates and that cells must somehow get rid of them. I propose that they do so by excreting DCC in extracellular vesicles.

PIP assumes near-perfect cellular repair systems that enable these cells to avoid the build-up of DCC. But can any repair system be perfect? If some DCC cannot be dissolved and recycled and hence accumulate, how could a repair system get rid of them? Almost the only possible answer is by expelling DCC from the cells by exocytosis.

Thus the two paradigms would converge if DCC are in fact expelled from ESC and cancerous cell lines in extracellular vesicles. For USP the expulsion of DCC would be seen as an excretory system, and for PIP a repair system. The effect would be the same.

After more than 130 years, it may soon be possible to resolve the debate between Maupas and Weismann. Maupas was right in thinking that senescence is inherent in all cells, and Weismann was right in thinking that some cell types in animals were special, able to avoid senescence in a way that is suppressed in most somatic cells. If this special repair system depends on the exocytosis of DCC, then we may soon arrive at a new synthesis that applies to all the kingdoms of life. I call this the cellular rejuvenation hypothesis: DCC build up in all cells as they age, but can be diluted either by growth and cell division, especially ACD and multiple fission or by the excretion of DCC in extracellular vesicles.

This cellular rejuvenation hypothesis is experimentally testable and in electronic supplementary material, appendix, I suggest nine new lines of empirical enquiry.

## Data Availability

This article has no additional data.

## References

[1] Maupas A. 1888 Recherches expérimentales sur la multiplications des infusoires ciliés. Arch. Zool. Exp. Gén., 2 **6**, 165-177.

[2] Weismann A. 1891 Essays upon heredity and kindred biological problems, Vol. I. Oxford, UK: Clarendon Press.

[3] Gladyshev VN. 2013 The origin of aging: imperfectness-driven non-random damage defines the aging process and control of lifespan. Trends Genet. **29**, 506-512. (10.1016/j.tig.2013.05.004) 23769208 PMC3804915

[4] Moger-Reischer R, Lennon JT. 2019 Microbial ageing and longevity. Nat. Rev. Microbiol. **17**, 679-690. (10.1038/s41579-019-0253-y) 31534207

[5] Currais A. 2016 The origin of life at the origin of ageing? Ageing Res. Rev. **35**, 297-300. (10.1016/j.arr.2016.10.007) 27818249

[6] Kirkwood T. 1977 Evolution of aging. Nature **270**, 301-304. (10.1038/270301a0) 593350

[7] Kirkwood TBL, Cremer T. 1982 Cytogerontology since 1881: a reappraisal of August Weismann and a review of modern progress. Hum. Genet. **60**, 101-121. (10.1007/BF00569695) 7042533

[8] Clegg RJ, Dyson RJ, Kreft JU. 2014 Repair rather than segregation of damage is the optimal unicellular aging strategy. BMC Biol. **12**, 52. (10.1186/s12915-014-0052-x) 25184818 PMC4243282

[9] Skloot R. 2019 The immortal life of Henrietta Lacks. London, UK: Picador.

[10] Nystrom T. 2005 Role of oxidative carbonylation in protein quality control and senescence. EMBO J. **24**, 1311-1317. (10.1038/sj.emboj.7600599) 15775985 PMC1142534

[11] Iuchi K, Takai T, Hisatomi H. 2021 Cell death via lipid peroxidation and protein aggregation diseases. Biology **10**, 399. (10.3390/biology10050399) 34064409 PMC8147787

[12] Finkel T, Holbrook NJ. 2000 Oxidants, oxidative stress and the biology of ageing. Nature **408**, 239-246. (10.1038/35041687) 11089981

[13] Roth LW, Polotsky AJ. 2012 Can we live longer by eating less? A review of caloric restriction and longevity. Maturitas **71**, 315-319. (10.1016/j.maturitas.2011.12.017) 22281163

[14] Protter DSW, Parker R. 2016 Principles and properties of stress granules. Trends Cell Biol. **26**, 668-679. (10.1016/j.tcb.2016.05.004) 27289443 PMC4993645

[15] Bard JAM, Ellen A, Goodall EA, Greene ER, Jonsson E, Dong KC, Martin A. 2016 Structure and function of the 26S proteasome. Annu. Rev. Biochem. **87**, 697-724. (10.1146/annurev-biochem-062917-011931) PMC6422034 29652515

[16] Maxwell BA, Gwon Y, Mishra A, Peng J, Nakamurake H, Zhang K, Kim HJ, Taylor JP. 2021 Ubiquitination is essential for recovery of cellular activities after heat shock. Science **372**, eabc3593. (10.1126/science.abc3593) 34739326 PMC8574219

[17] Gwon Y, Maxwell BA, Kolaitis RM, Zhang P, Kim HJ, Taylor JP. 2021 Ubiquitination of G3BP1 mediates stress granule disassembly in a context-specific manner. Science **372**, eabf6548. (10.1126/science.abf6548) 34739333 PMC8574224

[18] Zaffagnini G, Martens S. 2016 Mechanisms of selective autophagy. J. Mol. Biol. **428**, 1714-1724. (10.1016/j.jmb.2016.02.004) 26876603 PMC4871809

[19] Jimenez AJ, Perez F. 2017 Plasma membrane repair: the adaptable cell life-insurance. Curr. Opin Cell Biol. **47**, 99-107. (10.1016/j.ceb.2017.03.011) 28511145

[20] Igelmann S et al. 2021 A hydride transfer complex reprograms NAD metabolism and bypasses senescence. Mol. Cell **81**, 3848-3865. (10.1016/j.molcel.2021.08.028) 34547241

[21] López-Otín C, Blasco MA, Partridge L, Serrano M, Kroeme G. 2013 The hallmarks of aging. Cell **153**, 1194-1217. (10.1016/j.cell.2013.05.039) 23746838 PMC3836174

[22] Santos AL, Sinha S, Lindner AB. 2018 The good, the bad, and the ugly of ROS: new insights on aging and aging-related diseases from eukaryotic and prokaryotic model organisms. Oxidative Med. Cell. Longev. **2018**, 1941285. (10.1155/2018/1941285) PMC5878877 29743972

[23] Ogrodnik M, Salmonowicz H, Gladyshev VN. 2018 Integrating cellular senescence with the concept of damage accumulation in aging: relevance for clearance of senescent cells. Aging Cell **18**, e12841. (10.1111/acel.12841) 30346102 PMC6351832

[24] Brunk UTV. 1989 On the origin of lipofuscin: the iron content of residual bodies, and the relation of these organelles to the lysosomal vacuome. A study on cultured human glial cells. Adv. Exp. Med. Biol. **265**, 313-320. (10.1007/978-1-4899-5339-1_22) 2486159

[25] König J, Otta C, Hugoa M, Jung T, Bulteaub AL, Tilman Grunea T, Höhn A. 2017 Mitochondrial contribution to lipofuscin formation. Redox Biol. **11**, 673-681. (10.1016/j.redox.2017.01.017) 28160744 PMC5292761

[26] Brunk UTV, Terman A. 2002 Lipofuscin: mechanisms of age-related accumulation and influence on cell function. Free Radic. Biol. Med. **33**, 611-619. (10.1016/S0891-5849(02)00959-0) 12208347

[27] Moreno-Garcia A, Kun A, Calero O, Medina M, Calero M. 2018 A overview of the role of lipofuscin in age-related neurodegeneration. Front. Neurosci. **12**, 464. (10.3389/fnins.2018.00464) 30026686 PMC6041410

[28] Giaccone G, Orsi L, Cupidi C, Tagliavini F. 2011 Lipofuscin hypothesis of Alzheimer's disease. Dement Geriatr. Cogn. Disord. **1**, 292-296. (10.1159/000329544) PMC3235942 22545040

[29] Labuschagne CF, Brenkman AB. 2013 Current methods in quantifying ROS and oxidative damage in *Caenorhabdytis elegans* and other model organisms of aging. Ageing Res. Rev. **12**, 918-930. (10.1016/j.arr.2013.09.003) 24080227

[30] Ciaka K, Tymiński M, Gniazdowska A, Krasuska U. 2020 Carbonylation of proteins—an element of plant ageing. Planta **252**, 12. (10.1007/s00425-020-03414-1) 32613330 PMC7329788

[31] Sheldrake AR. 1974 The ageing, growth and death of cells. Nature **250**, 381-385. (10.1038/250381a0)

[32] Hirsch HR. 1978 The waste-product theory of aging: waste dilution by cell division. Mech. Aging Dev. **8**, 51-62. (10.1016/0047-6374(78)90006-4) 692175

[33] Erjavec N, Cvijovic M, Klipp E, Nyström T. 2008 Selective benefits of damage partitioining in unicellular systems and its effect on aging. Proc. Natl Acad. Sci. USA **105**, 18 764-18 769. (10.1073/pnas.0804550105) PMC2596250 19020097

[34] Moore DL, Jessberger S. 2017 Creating age asymmetry: consequences of inheriting damaged goods in mammalian cells. Trends Cell Biol. **27**, 82-92. (10.1016/j.tcb.2016.09.007) 27717533

[35] Rando TA. 2006 Stem cells, ageing and the quest for immortality. Nature **441**, 1080-1086. (10.1038/nature04958) 16810243

[36] Stern MM, Bickenbach JR. 2007 Epidermal stem cells are resistant to cellular aging. Aging Cell **6**, 439-452. (10.1111/j.1474-9726.2007.00318.x) 17635170

[37] Rujano MA et al. 2006 Polarized asymmetric inheritance of accumulated protein damage in higher eukaryotes. PLoS Biol. **4**, e417. (10.1371/journal.pbio.0040417) 17147470 PMC1750924

[38] Goodell MA, Rando TA. 2015 Stem cells and healthy aging. Science **350**, 1199-1204. (10.1126/science.aab3388) 26785478

[39] Lopez-Garcia C, Klein AM, Simons BD, Winton DJ. 2010 Intestinal stem cell replacement follows a pattern of neutral drift. Science **330**, 822-825. (10.1126/science.1196236) 20929733

[40] Johnston HE, Samant RS. 2021 Alternative systems for misfolding protein clearance: life beyond the proteasome. FEBS J. **288**, 4464-4487. (10.1111/febs.15617) 33135311

[41] Fuentealba LC, Elvers E, Geissert G, Taelman V, De Robertis EM. 2008 Asymmetric mitosis: unequal segregation of proteins destined for degradation. Proc. Natl Acad. Sci. USA **105**, 7732-7737. (10.1073/pnas.0803027105) 18511557 PMC2402384

[42] Yamashita YM. 2009 The centrosome and asymmetric cell division. Prion **3**, 84-88. (10.4161/pri.3.2.8821) 19458491 PMC2712604

[43] Ogrodnik M et al. 2014 Dynamic JUNQ inclusion bodies are asymmetrically inherited in mammalian cell lines through the asymmetric partitioning of vimentin. Proc. Natl Acad. Sci. USA **111**, 8049-8054. (10.1073/pnas.1324035111) 24843142 PMC4050583

[44] Katajisto P et al. 2015 Asymmetric apportioning of aged mitochondria between daughter cells is required for stemness. Science **348**, 340-343. (10.1126/science.1260384) 25837514 PMC4405120

[45] Wan Y, Finkel T. 2020 The mitochondrial regulation of stem cell aging. Mech. Aging Dev. **191**, 111334. (10.1016/j.mad.2020.111334) 32818514 PMC7541753

[46] Hayflick L. 1975 Cell biology of aging. Bioscience **25**, 629-637. (10.2307/1297030)

[47] Shay JW, Wright WF. 2000 Hayflick, his limit and cellular ageing. Nat. Rev. Mol. Cell Biol. **1**, 72-76. (10.1038/35036093) 11413492

[48] Balin AK, Fisher AJ, Anzelone M, Leong I, Allen RG. 2002 Effects of establishing cell cultures and cell culture conditions on the proliferative life span of human fibroblasts isolated from different tissues and donors of different ages. Exp. Cell Res. **274**, 275-287. (10.1006/excr.2002.5485) 11900488

[49] Shay JW, Wright WF. 2019 Telomeres and telomerase: three decades of progress. Nat. Rev. Genet. **20**, 299-309. (10.1038/s41576-019-0099-1) 30760854

[50] Schmutz I, de Lange T. 2016 Shelterin. Curr. Biol. **26**, R397. (10.1016/j.cub.2016.01.056) 27218840

[51] Aubert G, Lansdorp PM. 2008 Telomeres and aging. Physiol. Rev. **88**, 557-579. (10.1152/physrev.00026.2007) 18391173

[52] Donate LE, Blasco MA. 2011 Telomeres in cancer and ageing. Proc. R. Soc. B **366**, 76-84. (10.1098/rstb.2010.0291) PMC3001316 21115533

[53] Lu DH et al. 2019 Lysine demethylase 2A promotes the progression of ovarian cancer by regulating the PI3 K pathway and reversing epithelial–mesenchymal transition. Oncol. Rep. **41**, 917-927. (10.3892/or.2018.6888) 30483796 PMC6313075

[54] Wang J, Hannon GJ, Beach DH. 2000 Risky immortalization by telomerase. Nature **405**, 755-756. (10.1038/35015674) 10866187

[55] Miller DM, Thomas SD, Islam A, Muench D, Sedoris K. 2012 c-*Myc* and cancer metabolism. Clin. Cancer Res. **18**, 5546-5553. (10.1158/1078-0432.CCR-12-0977) 23071356 PMC3505847

[56] Huberman AD. 2020 Sight restored by turning back the epigenetic clock. Nature **588**, 34-36. (10.1038/d41586-020-03119-1) 33268872

[57] Ehrlich M. 2009 DNA hypomethylation in cancer cells. Epigenomics **1**, 239-259. (10.2217/epi.09.33) 20495664 PMC2873040

[58] Ding C, Schreiber SG, Roberts DR, Hamann A, Brouard JS. 2017 Post-glacial biogeography of trembling aspen inferred from habitat models and genetic variance in quantitative traits. Sci. Rep. **7**, 4672. (10.1038/s41598-017-04871-7) 28680120 PMC5498503

[59] Heidstra R, Sabatini S. 2014 Plant and animal stem cells: similar yet different. Nat. Rev. Mol. Cell Biol. **15**, 301-312. (10.1038/nrm3790) 24755933

[60] Pillitteri LJ, Guo X, Dong J. 2016 Asymmetric cell division in plants: mechanisms of symmetry breaking and cell fate determination. Cell Mol. Life Sci. **73**, 4213-4229. (10.1007/s00018-016-2290-2) 27286799 PMC5522748

[61] Smith GM. 1955a Cryptogamic botany: algae and fungi, vol 1. New York, NY: McGraw-Hill.

[62] Ivanov IN, Vítová M, Bišová K. 2019 Growth and the cell cycle in green algae dividing by multiple fission. Folia Microbiol. **64**, 663-672. (10.1007/s12223-019-00741-z) 31347103

[63] Bišova K, Zachleder V. 2014 Cell-cycle regulation in green algae dividing by multiple fission. J. Exp. Bot. **65**, 2585-2602. (10.1093/jxb/ert466) 24441762

[64] Smith GM. 1955b Cryptogamic botany: bryophytes and pteridophytes, vol. 2. New York, NY: McGraw-Hill.

[65] Steiner UK. 2021 Senescence in bacteria and its underlying mechanisms. Front. Cell Dev. Biol. **9**, 688915. (10.3389/fcell.2021.668915) PMC8249858 34222238

[66] Ackermann M. 2008 Bacteria as a new model system for aging studies: investigations using light microscopy. Biotechniques **44**, 564-567. (10.2144/000112829) 18476823

[67] Stewart EJ, Madden R, Paul G, Taddel F. 2005 Aging and death in an organism that reproduces by morphologically symmetric division. PLoS Biol. **3**, e45. (10.1371/journal.pbio.0030045) 15685293 PMC546039

[68] Lindner AB, Madden R, Demarez A, Stewart EJ, Tadei F. 2008 Assymmetric segregation of protein aggregates is associated with cellular aging and rejuvenation. Proc. Natl Acad. Sci. USA **105**, 3076-3081. (10.1073/pnas.0708931105) 18287048 PMC2268587

[69] Lapinska U, Glover G, Capilla-Lasheras P, Young AJ, Pagliara S. 2019 Bacterial ageing in the absence of external stressors. Phil. Trans. R. Soc. B **374**, 20180442. (10.1098/rstb.2018.0442) 31587633 PMC6792439

[70] Proenca AM, Rang CU, Qiu A, Shi C, Chao L. 2019 Cell aging preserves cellular immortality in the presence of lethal levels of damage. PLoS Biol. **17**, e3000266. (10.1371/journal.pbio.3000266) 31120870 PMC6532838

[71] Mortimer RK, Johnston JR. 1959 Life span of individual yeast cells. Nature **183**, 1751-1752. (10.1038/1831751a0) 13666896

[72] Spokoini R, Moldavski O, Nahmias Y, England JL, Schuldiner M, Kaganovich D. 2015 Confinement of organelle-associated inclusion structures mediates asymmetric inheritance of aggregated protein in budding yeast. Cell Rep. **2**, 738-747. (10.1016/j.celrep.2012.08.024) 23022486

[73] McFaline-Figueroa JR, Vevea J, Swayne TC, Zhou C, Liu C, Leung G, Boldogh IR, Pon LA. 2011 Mitochondrial quality control during inheritance is associated with lifespan and mother–daughter age asymmetry in budding yeast. Aging Cell **10**, 885-895. (10.1111/j.1474-9726.2011.00731.x) 21726403 PMC3173513

[74] Zhou C et al. 2014 Organelle-based aggregation and retention of damaged proteins in asymmetrically dividing cells. Cell **159**, 1-13. (10.1016/j.cell.2014.09.026) PMC6726438 25417105

[75] Higuchi-Sanabria R, Pernice WMA, Vevea JD, Dana M, Wolken A, Boldogh IR, Pon LA. 2014 Role of asymmetric cell division in lifespan control in *Saccharomyces cerevisiae*. Yeast Res. **14**, 1133-1145. (10.1111/1567-1364.12216) PMC4270926 25263578

[76] Saarikangas J, Caudron F, Prasad R, Moreno DF, Bolognesi A, Aldea M, Barral Y. 2017 Compartmentalization of ER-bound chaperone confines protein deposit formation to the aging yeast cell. Curr. Biol. **27**, 1-11. (10.1016/j.cub.2017.01.069) 28262489

[77] Coelho M et al. 2013 Fission yeast does not age under favorable conditions, but does so after stress. Curr. Biol. **23**, 1-9. (10.1016/j.cub.2013.07.084) 24035542 PMC4620659

[78] He C, Zhou C, Kennedy BK. 2018 The yeast replicative aging model. Biochem. Biophys. Acta **1864**, 2690-2696. (10.1016/j.bbadis.2018.02.023) 29524633

[79] de Kretser DM, Loveland KL, Meinhardt A, Simorangkhir D, Wreford N. 1998 Spermatogenesis. Hum. Reprod. **13**(Suppl. 1), 1-8. (10.1093/humrep/13.suppl_1.1) 9663765

[80] Wilson ZA, Zhang DB. 2009 From *Arabidopsis* to rice: pathways in pollen development. J. Exp. Bot. **60**, 1479-1492. (10.1093/jxb/erp095) 19321648

[81] Bastock R, Johnston DS. 2008 *Drosophila* oogenesis. Curr. Biol. **18**, R1082. (10.1016/j.cub.2008.09.011) 19081037

[82] Bufalino MR, DeVeale B, van der Kooy D. 2013 The asymmetric segregation of damaged proteins is stem cell-type dependent. J. Cell Biol. **201**, 523-530. (10.1083/jcb.201207052) 23649805 PMC3653353

[83] Yang WC, Shi DQ, Chen YH. 2010 Female gametophyte development in flowering plants. Annu. Rev. Plant Biol. **61**, 89-108. (10.1146/annurev-arplant-042809-112203) 20192738

[84] Liberti MV, Locasale JW. 2016 The Warburg effect: how does it benefit cancer cell? Trends Biochem. Sci. **41**, 211-218. (10.1016/j.tibs.2015.12.001) 26778478 PMC4783224

[85] Hanahan D, Weinberg RA. 2011 Hallmarks of cancer: the next generation. Cell **144**, 646-674. (10.1016/j.cell.2011.02.013) 21376230

[86] Reya T, Morrison SJ, Clarke MF, Weissman IL. 2001 Stem cells, cancer, and cancer stem cells. Nature **414**, 105-111. (10.1038/35102167) 11689955

[87] Batile E, Clevers H. 2017 Cancer stem cells revisited. Nat. Med. **10**, 1124-1134. (10.1038/nm.4409) 28985214

[88] Izumi H, Kaneko H. 2012 Evidence of asymmetric cell division and centrosome inheritance in human neuroblastoma cells. Proc. Natl Acad. Sci. USA **109**, 18 048-18 053. (10.1073/pnas.1205525109) 23064640 PMC3497809

[89] Desdin-Micó G, Mittelbrunn M. 2017 Role of exosomes in the protection of cellular homeostasis. Cell Adhes. Migr. **11**, 127-134. (10.1080/19336918.2016.1251000) PMC5351736 27875097

[90] Vagner T, Chin A, Mariscal J, Bannykh S, Engman DM, Di Vizio D. 2019 Protein composition reflects extracellular vesicle heterogeneity. Proteomics **19**, 1800167. (10.1002/pmic.201800167) PMC7521840 30793499

[91] Meckes DG, Raab-Traub N. 2011 Microvesicles and viral infection. J. Virol. **85**, 12 844-12 854. (10.1128/JVI.05853-11) PMC3233125 21976651

[92] Wiking L, Stagsted J, Björk L, Nielsen JH. 2004 Milk fat globule size is affected by fat production in dairy cows. Int. Dairy J. **14**, 909-913. (10.1016/j.idairyj.2004.03.005)

[93] Sheehan C, D'Souza-Schorey C. 2019 Tumor-derived extracellular vesicles: molecular parcels that enable regulation of the immune response in cancer. J. Cell Sci. **132**, jcs235085. (10.1242/jcs.235085) 31615844 PMC6826013

[94] Guenat D, Hermetet F, Prétet JL, Mougin C. 2017 Exosomes and other extracellular vesicles in HPV transmission and carcinogenesis. Viruses **9**, v9080214. (10.3390/v9080211) PMC5580468 28783104

[95] Takahashi A et al. 2017 Exosomes maintain cellular homeostasis by excreting harmful DNA from cells. Nat. Commun. **8**, 15287. (10.1038/ncomms15287) 28508895 PMC5440838

[96] Rajendran L, Honsho M, Zahn TR, Keller P, Geiger KD, Verkade P, Simons K. 2006 Alzheimer's disease β-amyloid peptides are released in association with exosomes. Proc. Natl Acad. Sci. USA **103**, 11 172-11 177. (10.1073/pnas.0603838103) PMC1544060 16837572

[97] Saman S et al. 2012 Exosome-associated tau is secreted in tauopathy models and is selectively phosphorylated in cerebrospinal fluid in early Alzheimer disease. J. Biol. Chem. **287**, 3842-3849. (10.1074/jbc.M111.277061) 22057275 PMC3281682

[98] Johnstone RM. 2005 Revisiting the road to the discovery of ectosomes. Blood Cells Mol. Dis. **14**, 214-219. (10.1016/j.bcmd.2005.03.002) 15885604

[99] Minciacchia VR, Freeman MR, Di Vizio D. 2015 Extracellular vesicles in cancer: exosomes, microvesicles and the emerging role of large oncosomes. Semin. Cell Dev. Biol. **40**, 41-51. (10.1016/j.semcdb.2015.02.010) 25721812 PMC4747631

[100] Hoshino A et al. 2020 Extracellular vesicle and particle biomarkers define multiple human cancers. Cell **182**, 1044-1061. (10.1016/j.cell.2020.07.009) 32795414 PMC7522766

[101] Kreger BT, Dougherty AL, Greene KS, Cerione RA, Antonyak MA. 2016 Microvesicle cargo and function changes upon induction of cellular transformation. J. Biol. Chem. **291**, 19 774-19 785. (10.1074/jbc.M116.725705) PMC5025668 27440046

[102] Kanada M, Bachmann MH, Contag CH. 2016 Signaling by extracellular vesicles advances cancer hallmarks. Trends Cancer **2**, 84-94. (10.1016/j.trecan.2015.12.005) 28741553

[103] Lin Y, Zhang C, Xiang P, Shen J, Sun W, Yu H. 2020 Exosomes derived from HeLa cells break down vascular integrity by triggering endoplasmic reticulum stress in endothelial cells. J. Extracell. Vesicles **9**, 1722385. (10.1080/20013078.2020.1722385) 32128072 PMC7034510

[104] Rang CU, Proenca A, Buetz C, Shi S. 2018 Minicells as a damage disposal mechanism in *Escherischia coli*. mSphere **3**, e00428-18. (10.1128/mSphere.00428-18) 30232168 PMC6147132

[105] Zeng X. 2007 Human embryonic stem cells: mechanisms to escape replicative senescence? Stem Cell Rev. **3**, 270-279. (10.1007/s12015-007-9005-x) 18026912

[106] Prasad SM et al. 2009 Continuous hypoxic culturing maintains activation of Notch and allows long-term propagation of human embryonic stem cells without spontaneous differentiation. Cell Prolif. **42**, 63-74. (10.1111/j.1365-2184.2008.00571.x) 19143764 PMC6496631

[107] Keeley TP, Mann GE. 2019 Defining physiological normoxia for improved translation of cell physiology to animal models and humans. Physiol. Rev. **161**, 234. (10.1152/physrev.00041.2017) 30354965

[108] Kaneko KJ, DePamphilis ML. 2013 TEAD4 establishes the energy homeostasis essential for blastocoel formation. Development **140**, 3680-3690. (10.1242/dev.093799) 23903192 PMC3742148

[109] Han C et al. 2016 Exosomes and their therapeutic potentials of stem cells. Stem Cells Int. **2016**, 7653489. (10.1155/2016/7653489) 26770213 PMC4684885

[110] Zhu Q et al. 2019 Embryonic stem cells-derived exosomes endowed with targeting properties as chemotherapeutics delivery vehicles for glioblastoma therapy. Adv. Sci. **6**, 1801899. (10.1002/advs.201801899) PMC6425428 30937268

[111] Schoenhals M, Kassambara A, De Vos J, Hose D, Moreaux J, Klein B. 2009 Embryonic stem cell markers expression in cancers. Biochem. Biophys. Res. Commun. **383**, 157-162. (10.1016/j.bbrc.2009.02.156) 19268426

[112] Kim J, Orkin SH. 2011 Embryonic stem cell-specific signatures in cancer: insights into genomic regulatory networks and implications for medicine. Genome Med. **3**, 75. (10.1186/gm291) 22126538 PMC3308030

[113] Mathieu J et al. 2011 HIF induces human embryonic stem cell markers in cancer cells. Cancer Res. **71**, 4640-4652. (10.1158/0008-5472.CAN-10-3320) 21712410 PMC3129496

[114] Pezzuto A, Carico E. 2018 Role of HIF-1 in cancer progression: novel insights. A review. Curr. Mol. Med. **6**, 343-351. (10.2174/1566524018666181109121849) 30411685

